# Potential for Anthropogenic Fin Damage to Affect Individual Responses to Prey in Bluegill Sunfish (*Lepomis macrochirus*): A New Hypothesis for Kinematic Studies

**DOI:** 10.1093/iob/obac050

**Published:** 2022-12-16

**Authors:** H E Cohen, W Ray, O H Hawkins, E A Kane

**Affiliations:** Department of Biology, Georgia Southern University, Statesboro, GA 30458, USA; Department of Biology, Georgia Southern University, Statesboro, GA 30458, USA; Department of Biology, University of Louisiana at Lafayette, Lafayette, LA 70504, USA; Department of Biology, Georgia Southern University, Statesboro, GA 30458, USA; Department of Biology, University of Louisiana at Lafayette, Lafayette, LA 70504, USA

## Abstract

In fishes, damage to important morphological structures such as fins through natural damage and anthropogenic factors can have cascading effects on prey capture performance and individual fitness. Bluegill sunfish (*Lepomis macrochirus*) are a common freshwater species in North America, are a model organism for performance studies, and often experience natural injuries. We opportunistically sampled two populations of fish in the lab to generate a hypothesis for the effect of sub-lethal fin damage resulting from the capture technique on kinematic performance during prey capture in bluegill. We found no statistical differences in mean prey capture kinematics or predator accuracy, but damaged fish used more variable kinematics and more readily struck at non-prey items. We suggest that a reduction in stability and individual consistency occurs as a result of fin damage. This difference could have consequences for higher-order ecological interactions such as competitive ability, despite a lack of apparent performance cost at the individual level, and deserves consideration in future studies of prey capture performance in fish.

## Introduction

Fish fins play important roles in the stabilization of the body, generating propulsive forces to power swimming, and performing ecologically relevant behaviors such as prey capture, predator avoidance, habitat navigation, and mating (Drucker and Lauder 2002; Standen and Lauder 2005). For instance, the caudal fin is used to generate thrust and assist with forward locomotion, the dorsal and anal fins aid in stability and thrust generation, and the pectoral fins are used for braking and maneuvering (Webb 1984; Drucker and Lauder 2005; Standen and Lauder 2005). However, injuries to these locomotor structures as a result of predation, intraspecific attacks, fishing, and disease can negatively impact locomotor performance (Webb 1977; Barthel et al. 2003; Sinclair et al. 2011; Fu et al. 2013). Fin damage may result in a reduction of fin area or tearing within the fin membrane and can have sub-lethal consequences, including reduced fin movement, decreased maneuverability and swimming performance, adoption of unnatural body positions to protect injured fins, increased susceptibility to predation, displacement into low-flow areas, and reduced feeding efficiency (Ellis et al. 2008; Sinclair et al. 2011). In some cases, damage to fins may also be lethal (Sinclair et al. 2011). While it is intuitive that consequences of fin damage may impact the locomotor system in some way, it is necessary to also consider the use of fins during dependent behaviors such as suction prey capture, when locomotion is used to approach and target prey (Kane et al. 2019).

During suction feeding, force is generated that results in a parcel of water in front of the predator's mouth that envelops the prey and pulls the prey into the predator's mouth (Ferry-Graham et al. 2003; Day et al. 2005; Holzman et al. 2007). However, suction is temporally and spatially constrained, and the predator needs to position itself close to the prey at the moment when the suction force is strongest to maximize accuracy and the chance of success (Higham et al. 2006; Holzman et al. 2012; Kane and Higham 2014). Therefore, damage to locomotor structures may negatively impact the timing and positioning of a strike, decreasing strike accuracy. For planktivore specialists such as bluegill that produce powerful but spatially constrained suction volumes that rely on an accurate approach, fin damage could have significant consequences for prey capture outcomes. In this way, accuracy connects locomotor and feeding performance to a whole-organism outcome (i.e., success or failure) (Drost 1987; Irschick et al. 2008).

**Fig. 1:**
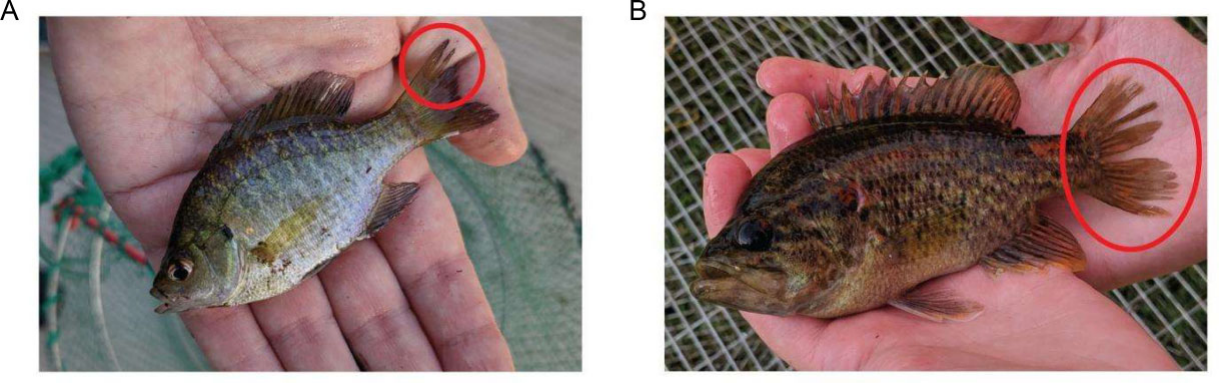
Fin damage is commonly observed in freshwater sunfish. (**A**) A bluegill, *Lepomis macrochirus*, caught in a minnow trap. (**B**) A warmouth, *Lepomis gulosus*, caught in a pinfish trap. Red circles highlight damage on the caudal fin of each fish. The type of damage shown here is similar to that observed as a result of capture using a cast net but is more extreme in the warmouth image.

Bluegill sunfish (*Lepomis macrochirus:* Rafinesque, 1819) are a common suction-feeding freshwater North American fish species that are often used as a model for understanding aquatic locomotion and suction prey capture performance (Wainwright et al. 2001; Drucker and Lauder 2002; Day et al. 2005; Kane and Higham 2014, 2020). Bluegill as well as other centrarchids are also known to experience injuries naturally and as a result of fishing interactions (Fig. 1). Higham et al. (2005b) examined the effect of partial ablation of pectoral fins on braking ability during prey capture in bluegill and found that compensatory behaviors may be able to overcome the negative effects of damage. However, the cascading effect of damage on feeding movements and prey capture outcomes was not examined.

In our exploratory study, we take advantage of the unexpected presence of fin damage in a laboratory population of bluegill captured using a monofilament cast net. Behaviors of these fish are contrasted with a control group that was independently and subsequently captured using a boat electrofisher. We develop a new hypothesis to describe how the recent presence of fin damage, resulting from a widely used collection technique, can affect prey capture outcomes. Since bluegill rely on a coordinated locomotor approach during prey capture (Kane and Higham 2020), we expect that fin damage can negatively affect approach and capture kinematics, predator accuracy, and success rate.

## Materials and methods

### Fish collection and husbandry

Independently sampled populations of juvenile bluegill were collected using two methods from Richmond Hills Hatchery: on June 15, 2018, using a monofilament cast net, which resulted in the monofilament getting caught between the fin rays and causing damage similar to that in Fig. 1 (*n* = 6, mean SL = 8.53 ± 0.75 cm), or on October 29, 2018, using a boat electrofisher, which resulted in stunned fish being collected with a mesh dipnet that did not damage fins (*n* = 6, SL = 10.02 ± 0.79 cm). Fish were returned to the lab, where they were housed individually or in pairs separated by a plastic divider in 38–76 L tanks, depending on space constraints. Holding tanks contained conditioned tap water and were exposed to overhead fluorescent lighting on a 12:12 h light: dark cycle and maintained at 24°C. Fish were fed daily with frozen/commercial shrimp or bloodworms. Since fish captured *via* cast net were initially deemed unusable, fin damage was not documented, and fish differed in their recovery duration at the time of filming. However, all damaged fish experienced one or more recent fin damage events (tears or missing pieces) prior to filming as a result of the collection technique, whereas the healthy fish did not. Our comparison of fish with or without damage is similar in design to previous work examining the effect of hook-and-line capture on feeding kinematics (Thompson et al. 2018).

Fish care and husbandry follow previous studies using bluegill (Kane and Higham 2014, 2020). Fish were tagged using visible elastomer implant tags (Northwest Marine Technology Inc., Shaw Island, WA, USA) to facilitate the identification of individual fish. These tags are small relative to fish size and do not affect fish movement or function (Dewey and Zigler 1996). Fish were allowed to recover for at least 1 week to ensure tag retention and animal recuperation (Wilson and Godin 2009). The care and use of experimental animals complied with the Institutional Animal Care and Use Committee animal welfare laws, guidelines, and policies as approved by Georgia Southern University (IACUC #18021).

### Experimental design

To quantify kinematic performance during prey capture, individual bluegill were recorded with a high-speed camera (Edgertronic SC1, Sanstreak Corp., San Jose, CA, USA) during attempts to capture non-evasive bloodworm prey. Prior to filming, fish were starved for up to 48 h to motivate feeding events. Each fish was transferred to a 75 L filming tank containing freshwater from the same source as holding tanks, with a working section of 53 × 32 × 29 cm, and given at least 3 h to acclimate prior to data collection. Thawed prey was introduced 1–2 at a time using a pipette. Fish were recorded using visible light illumination in the lateral view at 500 fps. Up to seven capture attempts were recorded on the same day, and fish were recorded over 2–3 days of trials. All saved videos (128 trials) were used to determine capture success rates; up to five videos per fish were selected for kinematic analysis when available (58 trials), including up to one missed capture attempt per fish to maximize the kinematic variation observed within fish. Most damaged fish trials were filmed between July 1, 2018 and October 15, 2018 (four trials for one fish were filmed between November 2, 2018 and December 8, 2018). All healthy fish trials were filmed between December 14, 2018 and December 28, 2018.

### Kinematic analysis

**Fig. 2:**
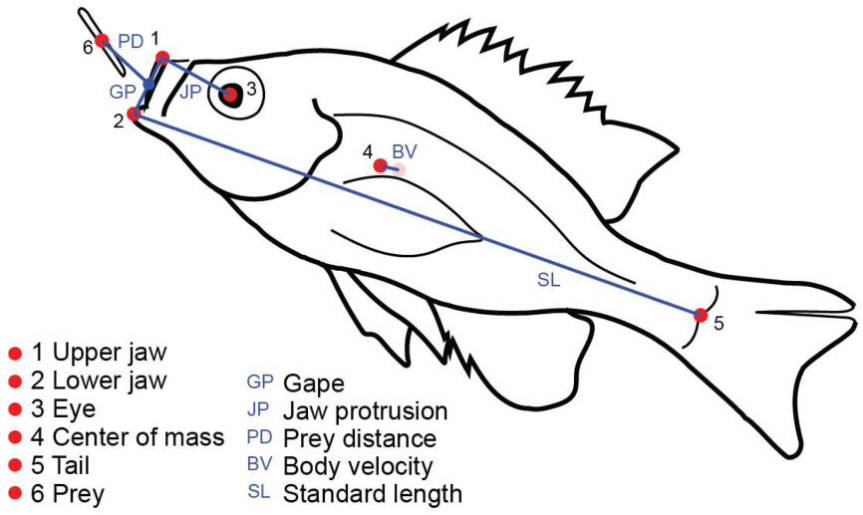
Trace of a still image from an analyzed video shows six digitized points (red circles) used to calculate five distances (blue lines) and extract kinematic variables. Representative fin damage is shown on the caudal fin, but fin damage was not controlled and may have been present to varying degrees on any fin.

Six points describing the movement of the jaws, body, and prey (Fig. 2) were digitized in each video using the DLTdv5 package (Hedrick 2008) in MATLAB (MATLAB 2017b; Mathworks, Natick, MA, USA). These landmarks included the antero-dorsal tip of the premaxilla (upper jaw), the antero-ventral tip of the dentary (lower jaw), the center of the pupil (eye), the approximate midpoint along the line between the anterior insertion of the dorsal and pelvic fins (approximate center of mass), the midpoint along the posterior margin of scales on the caudal peduncle (tail), and the center of mass of the prey. Points were digitized for each trial once jaw motion was initiated and ended once jaws closed and prey was captured. The prey was digitized from the beginning of the sequence until it entered the mouth of the fish and was no longer visible. Points were smoothed using a quintic spline (MATLAB, custom script) to minimize digitizing errors. Tolerance values were chosen manually, and smoothed traces were visually compared to raw traces to ensure the accuracy of smoothed points relative to movements observed in the video.

Smoothed landmark XY values were used to calculate kinematics, including gape distance (distance between jaws), jaw protrusion distance (distance between the upper jaw and eye), prey distance (distance between the prey and the midpoint of the gape), body displacement (distance traveled by the center of mass between subsequent frames), and standard length (SL) (distance between the closed lower jaw and the tail point). Gape opening, jaw protrusion, and body velocities were calculated as the change in each linear distance divided by the time elapsed between subsequent frames (0.002 s). Body acceleration was calculated as the difference in body velocity between frames divided by the elapsed time. The tail point was digitized only for a single frame, corresponding to when the lower jaw was closed and the fish's body was fully extended (not curved), resulting in a single measure of SL for the trial. All other measures were calculated for the duration of the trial to produce kinematic traces through time.

Following prior work, peak gape was manually chosen as the nearest value to 95% peak gape, and mouth opening and closing were chosen as the nearest values to 20% peak gape (Sanford and Wainwright 2002). Exceptions occurred when the mouth was held open at >20% peak gape, in which case gape velocity was used to determine the time at which mouth opening began to increase or ceased to decrease (determined by visual inspection of plots for each trial). These values were used to determine the time to opening (duration from opening to peak gape) and time to closing (duration from peak gape to closing). Following the determination of the time of peak gape, trials were re-centered such that timing events prior to peak gape were negative. This analysis resulted in a total of 14 kinematic variables describing the locomotor approach and prey capture.

In addition to kinematics, measures describing the outcome of these kinematics were also calculated. Capture success/miss rates were quantified using the subset of trials analyzed for kinematics as well as all additional recorded trials. Missed attempts included those where the prey did not cross the boundary formed by the upper and lower jaws during the first strike attempt. Three metrics of predator accuracy were calculated using two kinematic variables from the subset of analyzed videos described above, as follows (Kane and Higham 2014): peak gape and body velocity at the time of peak gape were used to estimate the volume of ingested water, ingested volume height to length ratio, and accuracy index (AI). In addition, the linear distance between predator and prey has also been used as a measure of predator accuracy (O'Brien 1979; McLaughlin et al. 2000; Ioannou et al. 2012), and we used the predator–prey distance at the time of mouth opening as an alternative, fourth, metric of accuracy.

### Statistical analysis

Extracted variables were analyzed separately as three datasets containing either kinematic traits (locomotor approach + feeding), accuracy traits, or success rates. Both kinematic and accuracy datasets were analyzed with general linear mixed models (GLMMs) to assess differences between healthy and damaged populations for each trait. Assumptions of linearity were visually confirmed using a quantile-quantile plot, homogeneity of variance was visually confirmed using a residual plot and quantitatively using Levene's test, and extreme outliers were identified. No outliers were removed for this analysis. Models were run in two ways: first, with population (healthy vs. damaged) as a fixed effect and individual as a random effect, and second, including SL as an additional fixed effect. Differences between these models add insight into the effect of body size on kinematics and were compared using Akaike information criterion (AIC) and Bayesian information criterion (BIC) values to select the best model for detecting differences between healthy and damaged individuals (Table 1). Differences between success rates were assessed using a Chi-square (*χ*^2^) analysis.

**Table 1:** Model selection of GLMM with and without SL as a fixed effect for each kinematic variable

Kinematic variable	Model	AIC	BIC	Log likelihood	Deviance	*χ* ^2^	df	*P*-value
Peak gape (cm)	1	–81.89	–73.64	44.94	–89.89	10.84	1	*P* < 0.0001*
	**2**	–90.72	–80.42	50.36	–100.72			
Peak protrusion (cm)	1	–158.49	–150.25	83.244	–166.49	5.09	1	0.02*
	**2**	–161.57	–151.27	85.788	–171.57			
Protrusion velocity (cm/s)	**1**	420.55	428.79	–206.28	412.55	0.19	1	0.66
	2	422.36	432.66	–206.18	412.36			
Time of peak protrusion (ms)	**1**	419.91	428.15	–205.96	411.91	1.22	1	0.27
	2	420.69	430.99	–205.34	410.69			
Duration of mouth opening (ms)	**1**	467.65	475.89	475.89	–229.82	0.16	1	0.69
	2	469.49	479.79	479.79	–229.75			
Duration of mouth closing (ms)	**1**	536.24	544.48	–264.12	528.24	0.38	1	0.54
	2	537.86	548.16	–263.93	527.86			
Maximum body velocity (cm/s)	**1**	433.39	441.63	–212.69	425.39	0.0001	1	0.99
	2	435.39	445.69	–212.69	425.39			
Time of maximum velocity (cm/s)	**1**	508.90	517.14	–250.45	500.90	0.14	1	0.70
	2	510.76	521.06	–250.38	500.76			
Velocity at peak gape (cm/s)	**1**	413.61	421.86	–202.81	405.61	0.33	1	0.57
	2	415.28	425.59	–202.64	405.28			
Acceleration at peak gape (cm/s^2^)	**1**	955.97	964.21	–473.99	947.97	1.00	1	0.32
	2	956.97	967.27	–473.48	946.97			
Accuracy Index (AI)	**1**	19.444	27.686	–5.7222	11.444	0.29	1	0.59
	2	21.154	31.457	–5.5772	11.154			
Height to length ratio of ingested volume	**1**	–140.87	–132.63	74.436	–148.87	0.57	1	0.45
	2	–139.44	–129.14	74.719	–149.44			
Ingested volume (cm^3^)	**1**	258.21	266.46	–125.11	250.22	2.68	1	0.10
	2	257.53	267.83	–123.77	247.53			
Predator-prey distance at mouth opening (cm)	**1**	77.44	85.68	–34.72	69.44	0.74	1	0.39
	2	78.70	89.00	–34.35	68.70			

Reported parameters for each model include AIC, BIC, log likelihood, deviance, Chi-square (*χ*^2^), degrees of freedom (df), and the *P*-value. The reduced model (Model 1) uses the following formula: *kinematic trait∼Population + (1|Individual).* The full model (Model 2) uses the following formula: *kinematic trait∼Population + SL + (1|Individual).* Population is the primary fixed effect that accounts for the observations belonging to healthy or damaged fish. Individual is included as a random effect to control for any influence of individual variation. A third model including the interaction of standard length and population as a fixed effect (SL: Population) was compared to Model 2 and found to not significantly differ, therefore we proceeded with Model 2 as the full model. When an Analysis of Variance (ANOVA) returned a *P*-value below the cutoff (0.05), the full model was used for univariate analysis.

Significance is denoted by an asterisk (*), and selected models are bolded.

In addition to these univariate tests, differences between healthy and damaged populations for the kinematic dataset only were also assessed using a quadratic discriminant analysis (QDA). To account for the few variables that expressed effects of body size, multivariate analyses used residuals from regressions of log_10_-transformed traits against log_10_-transformed SL. The QDA assumed unequal prior probabilities, proportional to trial occurrence (healthy = 0.48, damaged = 0.52). A quadratic model assuming different covariances between groups provided a better fit than a linear model assuming similar covariances and was chosen for interpretation (linear: number misclassified = 20, 34.5%, entropy *R*^2^ = 0.162, –2LogLikelihood = 67.37; quadratic: number misclassified = 8, 13.8%, entropy *R*^2^ = 0.711, –2LogLikelihood = 23.21).

Finally, to further summarize and explore potential patterns in kinematic traits within and between populations, multivariate patterns in residual kinematic traits were visualized using a principal component analysis (PCA). Principal component (PC) scores were then used to assess within-individual distance and between-individual distance (similar to Araujo et al. 2007). Within-individual distance was determined by calculating the mean linear distance between the mean XY position on the PCA and all trials for each individual. Between individual distance was calculated using the linear distance between individual mean XY positions on the PCA. Differences between populations for PC scores, consistency, and overlap were assessed using t-tests. All statistical analyses were completed using R version 1.4.1 and the following packages: “lme4,” “rstatix,” “prcomp,” and “MASS” (R Core Team 2021), with the exception of the QDA, which was performed in JMP Pro 15 (SAS Institute, Inc., Cary, NC, USA).

## Results

Overall, kinematic differences between populations of bluegill with either healthy or damaged fins were slight or not statistically differentiated. Most kinematics associated with mouth movements (feeding) were smaller or earlier in damaged fish by 7% or more, and most kinematics associated with body movements (swimming) were larger in damaged fish by 5% or more (Table 3). None of these results were significantly different between populations using univariate GLMMs (Table 2), apart from acceleration at peak gape if we used a less conservative cutoff of *P* < 0.1 (Table 3). As with most univariate analyses, differences between populations were similarly absent when using a multivariate QDA (F_10,47_ = 1.1977; *P* = 0.3169, Table 4). The minimal effects of fin damage that we observed were not likely the result of including variation due to missed capture attempts (two healthy, three damaged), since a reduced dataset that only included three trials per fish (the minimum number available across all fish; zero healthy, two damaged misses) showed similar trends. Although fish with intact fins were 15% larger than those with damaged fins, only peak gape and peak protrusion had significant effects on body size (Table 2).

**Table 2:** Comparison of GLMM with and without SL as a fixed effect for each kinematic variable

Kinematic variable	Model	Predictors	Estimates	Error	df	*t*-value	*P*-value by estimate
Peak gape (cm)	1	Intercept	0.89	0.04	10.29	21.784	*P* < 0.0001**
		Population	–0.0021	0.58	0.58	–0.036	0.97
	**2**	Intercept	0.14	0.23	52.36	0.63	0.53
		Population	0.10	0.06	14.26	1.58	0.14
		SL	0.08	0.02	0.23	3.38	*P* < 0.01**
Peak protrusion (cm)	1	Intercept	0.38	0.02	9.85	21.51	*P* < 0.0001**
		Population	–0.08	0.05	9.61	–3.08	0.01**
	**2**	Intercept	0.12	0.12	42.01	1.04	0.30
		Population	–0.04	0.03	15.36	–1.53	0.15
		SL	0.03	0.01	42.65	2.20	0.03**
Peak protrusion velocity (cm/s)	**1**	Intercept	18.54	2.36	10.53	7.85	*P* < 0.0001**
		Population	–1.37	3.31	10.25	–0.42	0.69
	2	Intercept	11.60	17.93	40.30	0.65	0.52
		Population	–0.44	15.85	15.85	–0.11	0.92
		SL	0.72	1.85	40.79	0.39	0.70
Time of peak protrusion (ms)	**1**	Intercept	–0.51	3.43	9.75	–0.15	0.89
		Population	–2.16	4.84	0.62	–0.45	0.67
	2	Intercept	19.40	18.62	53.81	1.04	0.30
		Population	–4.80	5.43	13.40	–0.88	0.39
			–2.10	1.90	54.63	–1.09	0.28
		SL					
Duration of mouth opening (ms)	**1**	Intercept	43.68	3.48	10.01	12.56	*P* < 0.0001**
		Population	–4.68	4.87	9.72	–0.96	0.36
	2	Intercept	29.57	27.04	39.48	1.09	0.28
		Population	–2.81	6.25	14.15	–0.45	0.66
		SL	1.47	2.78	40.07	0.53	0.60
Duration of mouth closing (ms)	**1**	Intercept	78.87	7.19	10.34	10.97	*P* < 0.0001**
		Population	–17.00	10.10	10.11	–1.68	0.12
	2	Intercept	109.72	49.87	46.41	2.20	0.03**
		Population	–21.14	12.24	15.42	–1.73	0.10
		SL	–3.21	5.13	47.30	–0.63	0.53
Maximum velocity (cm/s)	**1**	Intercept	26.73	2.84	10.46	9.41	*P* < 0.0001**
		Population	1.39	3.99	10.21	0.35	0.74
	2	Intercept	28.26	20.44	44.21	1.38	0.17
		Population	1.18	4.89	15.44	0.24	0.81
		SL	–0.16	2.10	44.97	–0.08	0.95
Time of maximum velocity (cm/s)	**1**	Intercept	–22.95	3.66	9.33	–6.28	*P* < 0.0001**
		Population	–7.26	5.09	8.95	–1.43	0.19
	2	Intercept	–7.14	34.65	23.16	–0.21	0.84
		Population	–9.34	7.13	12.78	–1.31	0.21
		SL	–1.65	3.57	23.00	–0.46	0.65
Velocity at peak gape (cm/s)	**1**	Intercept	19.66	2.45	10.43	8.04	*P* < 0.0001**
	2	Population	2.28	3.44	10.18	0.66	0.52
		Intercept	11.94	17.17	43.47	0.70	0.49
		Population	3.31	4.09	14.91	0.81	0.43
		SL	0.80	1.77	44.24	0.45	0.65
Acceleration at peak gape (cm/s^2^)	**1**	Intercept	62.52	164.79	56.00	0.38	0.71
		Population	–415.89	229.13	56.00	–1.82	0.07*
	2	Intercept	–1445.90	1548.50	55.00	–0.93	0.36
		Population	–209.10	311.60	55.00	–0.67	0.51
		SL	156.2	159.40	55.00	0.98	0.33
Accuracy index (AI)	**1**	Intercept	0.71	0.05	56.00	13.91	*P* < 0.0001**
		Population	–0.03	0.07	56.00	–0.39	0.70
	2	Intercept	0.466	0.49	55.00	0.95	0.35
		Population	0.01	0.98	55.00	0.07	0.94
		SL	0.03	0.05	55.00	0.53	0.60
Height to length ratio of ingested volume	**1**	Intercept	1.00	0.02	10.41	62.28	*P* < 0.0001**
		Population	–0.01	0.02	10.06	–0.61	0.56
	2	Intercept	0.88	0.14	33.60	6.36	*P* < 0.0001**
		Population	0.003	0.03	14.06	0.10	0.92
		SL	0.01	0.01	33.84	0.91	0.37
Ingested volume (cm^3^)	**1**	Intercept	5.86	0.75	10.34	7.85	*P* < 0.0001**
		Population	0.32	1.05	10.16	0.30	0.77
	2	Intercept	–1.11	4.50	49.59	49.59	0.81
		Population	1.25	1.16	15.07	15.07	0.30
		SL	0.72	0.46	50.62	50.62	0.12
Predator–prey distance at mouth opening (cm)	**1**	Intercept	1.68	0.12	10.34	13.72	*P* < 0.0001**
		Population	–0.26	0.17	10.06	–1.51	0.16
	2	Intercept	0.94	0.93	39.72	1.01	0.32
		Population	–0.16	0.21	15.48	–0.75	0.46
		SL	0.08	0.10	40.21	0.81	0.42

Reported parameters include the predictors and intercepts, coefficient estimates, error, degrees of freedom (df), *t-*values, and *P*-values for the contribution of the predictor to the model. The reduced model (Model 1) uses the following formula: *kinematic trait∼Population + (1|Individual).* The full model (Model 2) uses the following formula: *kinematic trait∼Population + SL + (1|Individual).* Population is the primary fixed effect that accounts for the observations belonging to healthy or damaged fish. Individual is included as a random effect to control for any influence of individual variation.

Significance is denoted by two asterisks (**) using a traditional cutoff of 0.05 or one asterisk (*) using a modified cutoff of 0.1.

The bolded model number is showing which quadratic model, assuming different covariances between groups, is a better fit.

**Table 3:** Summary statistics for all kinematic variables (feeding, swimming, and accuracy) by population.

Variable	Healthy	Damaged	% Difference
Kinematics			
Peak gape (cm)	0.89 ± 0.15	0.89 ± 0.10	0
Peak protrusion (cm)	0.38 ± 0.08	0.30 ± 0.04	–21.05
Peak protrusion velocity (cm/s)	18.58 ± 7.16	17.17 ± 10.23	–7.59
Time of peak protrusion (ms)	0.36 ± 11.98	–2.67 ± 7.34	–841.67
Duration of mouth opening (ms)	43.29 ± 12.13	39.0 ± 14.21	–9.91
Duration of mouth closing (ms)	78.36 ± 27.29	61.87 ± 22.17	–21.04
Maximum body velocity (cm/s)	26.65 ± 7.58	28.11 ± 11.98	5.48
Time of maximum velocity (ms)	–22.86 ± 19.33	–30.20 ± 17.85	32.11
Velocity at peak gape (cm/s)	19.76 ± 7.92	21.94 ± 9.79	11.03
Acceleration at peak gape (cm/s^2^)	65.52 ± 806.27	–353.38 ± 929.0	–639.35
Accuracy			
Accuracy index (AI)	0.71 ± 0.34	0.69 ± 0.18	–2.82
Height to length ratio of ingested volume	1.00 ± 0.6	0.99 ± 0.08	–1.00
Ingested volume (cm^3^)	5.88 ± 2.63	6.18 ± 2.09	5.10
Predator-prey distance at mouth opening (cm)	1.67 ± 0.49	1.42 ± 0.43	–14.97

Data are reported as mean ± SD of all observations from healthy (*n* = 28), and damaged (*n* = 30) trials. Percent difference represents the change observed in the damaged fish compared to healthy fish.

**Table 4:** Quadratic discriminant analysis coefficients for swimming and feeding kinematic variables.

Kinematic variables	Discriminant axis 1
Peak gape	**1.338**
Duration of mouth opening	0.035
Duration of mouth closing	0.021
Peak protrusion	**3.627**
Peak protrusion velocity	0.055
Time of peak protrusion	0.072
Velocity at peak gape	–0.120
Maximum velocity	0.050
Time of maximum velocity	0.048
Acceleration at peak gape	<0.0001

Data used in the analysis include observations from six healthy (28 trials) and six damaged (30 trials) individuals regressed against SL. Coefficients are bolded and considered significant contributors to the axis when their magnitude is greater than | 1 |.

For accuracy and success, which represent the functional outcomes of capture kinematics, differences between populations were similarly slight and statistically indistinguishable (Table 3, * χ*^2^_1_ = 0.28; *P* = 0.60). Bluegill with damaged fins ingested nearly 5% more water during prey capture and showed a 2.8% decrease in AI and an 86% increase in missed captures compared to fish with healthy fins. Decreased accuracy may have contributed to the concomitant increase in missed capture attempts in damaged fish (damaged fish 5/60 trials, 8.3%; healthy fish 3/67 trials, 4.5%). Notably, only fish with damaged fins attempted to capture bubbles ejected from the pipette, instead of prey. If these trials are counted as missed attempts, then the rate of missed captures increases to 10% in this group.

**Fig. 3:**
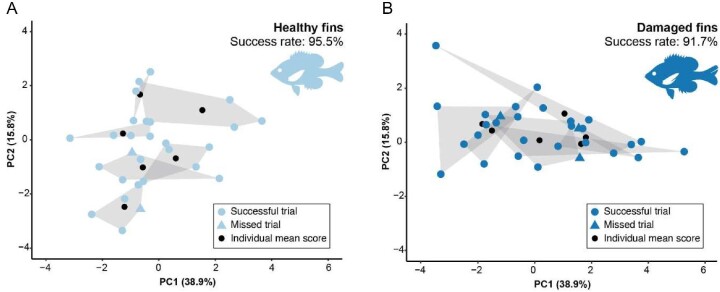
Principal component analysis shows variation among kinematic variables between populations. Individual hulls and means are shown for fish with (**A**) intact fins (light blue) or (**B**) damaged fins (dark blue). A single analysis was performed, but the plot was replicated to show each population.

Despite a lack of differentiation using tests explicitly designed to test our hypothesis, analyses using PCA scores revealed other areas of potential divergence between groups (Fig. 3). Two principal component axes explained 54.7% of the total variation in kinematics (Table 5) and were explained primarily by body and jaw approach speeds (also called ram speeds, PC1) or gape and protrusion magnitude and the timing of peak protrusion (PC2, Table 5). These axes can be summarized as roughly representing approach (PC1) or feeding (PC2)-related traits. Differences between scores for each axis were only apparent along PC2 when using a relaxed cutoff of *P* < 0.1 (Table 5). Therefore, locomotor approach traits comprise more of the kinematic variation (PC1), but less prominent feeding traits (PC2) may better represent the marginal divergence in kinematics between healthy and damaged populations. Interestingly, the variables explaining PC2 are also the 3 strongest coefficients in the QDA (Table 4), further corroborating the importance of these kinematics in distinguishing a minor degree of divergence. Additionally, healthy individuals appear to use kinematics that occupy more discreet space (Fig. 3A) compared to fish that experienced prior fin damage as a result of capture (Fig. 3B). Damaged individuals were much less consistent within individuals (116% greater mean distance among trials) and showed more overlap between individuals (1.7% smaller mean distance) than healthy fish (Fig. 3B).

**Table 5:** Loadings of principal components for feeding and swimming variables.

Kinematic variable	PC 1	PC 2
Peak gape	0.20	**0.59**
Duration of mouth opening	**–0.42**	0.21
Duration of mouth closing	–0.28	0.07
Peak protrusion	0.03	**–0.50**
Peak protrusion velocity	**0.42**	–0.15
Time of peak protrusion	–0.21	**–0.53**
Velocity at peak gape	**0.46**	0.05
Maximum velocity	**0.42**	–0.02
Time of maximum velocity	0.30	–0.19
Acceleration at peak gape	–0.07	–0.03
Percentage of variance by component	38.9%	15.8%
Total percentage of variation	38.9%	54.7%

Data used in the analysis include observations from six healthy (*n* = 28 trials) and six damaged (*n* = 30 trials) individuals regressed against SL. Principal component (PC) loadings indicate the contribution of each variable to each PC. Loadings are bolded and considered significant contributors to a PC when their magnitude is greater than | 0.3 |.

## Discussion

Overall, we found little support for the effects of prior recent fin damage on capture kinematics, predator accuracy, and success rate, contrary to our expectations. However, damaged fish relied on relatively less stable and predictable movements and showed reduced discretion of prey suitability. Damaged fish tended to approach prey using an earlier, faster burst of body ram that transitioned into deceleration at the time of peak gape, coupled with an earlier but less extensive jaw protrusion and a shorter mouth opening duration. Their brevity of movement coupled with deceleration might be used to regain stability, but may come at the cost of slightly lower accuracy and success. Healthy individuals, however, may use more predictable movements, allowing them to open their mouths slightly farther from the prey and maintain acceleration at peak gape, resulting in greater accuracy and success. We hypothesize that instability is likely exacerbated with more extreme damage, when kinematic differences may be more likely.

Alternatively, the minor degree of damage and kinematic instability observed here may not be the ecologically relevant outcome of fin damage, and capture with a cast net may be detrimental in other less obvious ways. Previously damaged fish lack the individualized capture strategies observed in healthy fish. Therefore, the consequence of fin damage is not necessarily in an individual's ability to capture prey, but rather in their ability to produce consistent kinematics. We hypothesize that the cost of decreased individualization is in the ability to compete in resource-limited environments. This novel insight describes the impact of locomotor form and function on secondary behaviors such as prey capture that deserves further attention.

Anthropogenic catch-and-release interactions can impose physiological stress and physical damage that have negative consequences for post-release behaviors (Thompson et al. 2018; LaRochelle et al. 2022), and capture with a cast net may be similarly detrimental if damage limits success in a more extreme or competitive context. However, bluegill are not usually the target species for this capture method, and it is unclear whether similar effects would be observed in non-model species. Therefore, further work assessing the types and extent of impacts on bluegill and other species of fish is necessary moving forward.

### Sample representation

Several limitations may have weakened our ability to decipher differences between fish that had healthy and previously damaged fins. Observation of videos (which only show one side of the fish and do not necessarily show fins in the protracted position) combined with our memory of fish indicated that damage was limited to 1–2 fins with 2–3 tears or rips across fins. Therefore, the degree of damage our fish received was likely relatively benign. Additionally, fins were at varying stages of healing depending on the individual (Johnson and Denton 1977; Böckelmann et al. 2010; Allen et al. 2018), and preliminary respirometry analyses (unpublished data) corroborated this effect. As a further limitation to our ability to detect kinematic differences, we chose a non-evasive prey type, which is less challenging than evasive or other prey types (Coughlin and Strickler 1990; Norton 1991; Svanbäck and Eklöv 2003; Kane and Higham 2014). Therefore, we likely underestimated performance costs. Further, without controlled damage and repeated measures across individuals, the link between observed damage and performance costs can only be indirect.

Despite these limitations, we suggest that our sample is broadly representative and our conclusion is appropriate. Since we do not observe statistically significant differences in mean kinematics, these traits likely do not depend on the degree or duration of damage within the range of what our fish experienced. On the other hand, we do observe differences between populations at the level of individualized responses, suggesting that uncontrolled factors such as the severity or duration of damage are less important than simple exposure to damage. Further, if variation in uncontrolled traits was responsible for observed differences among individuals, we would expect to see individualized kinematic responses in damaged fish, but the opposite is true. Interestingly, a lack of differentiation in approach and capture kinematics was also found between pelagic and littoral bluegill ecomorphs (Moran et al. 2018), suggesting that divergence in prey capture performance is not necessary for fish to experience ecological divergence.

**Fig. 4:**
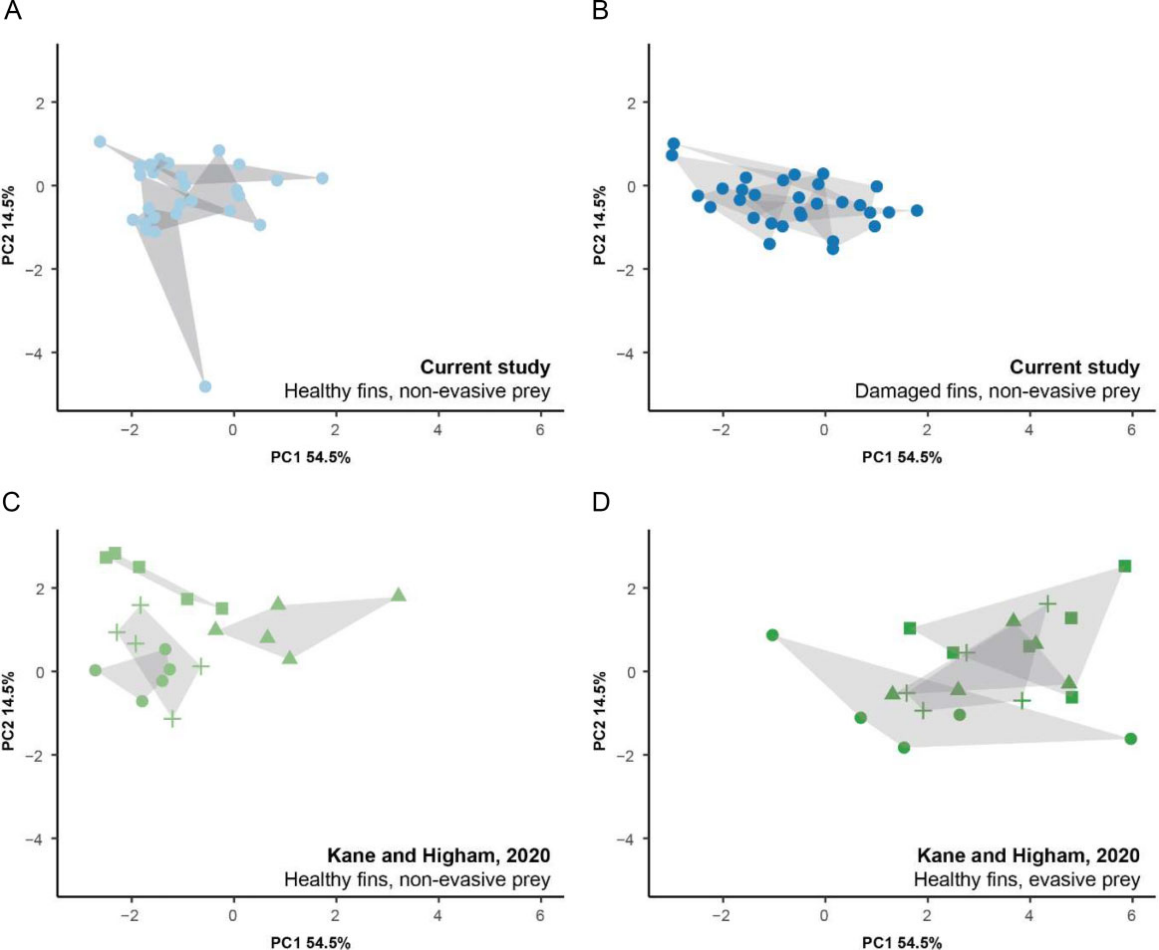
Principal component analysis of a subset of homologous data from the current study (*n* = 6) combined with previously collected bluegill data (*n* = 4; Kane and Higham 2020). (**A**) Fish with healthy fins capturing non-evasive prey, current study; (**B**) Fish with damaged fins capturing non-evasive prey, current study; (**C**) Fish with healthy fins capturing non-evasive prey, previous study; (**D**) Fish with healthy fins capturing evasive prey, previous study. A single analysis was performed, but the plot was replicated to show each population. Individuals from the prior study were used in both treatments and are differentiated using alternative shapes. Individual hulls are shown with gray shaded polygons. In both studies, challenges to prey capture, such as fin damage or evasive prey, result in a loss of individual differentiation.

To explore the representation of our sample, we directly compared a subset of homologous kinematic results to previously published prey capture trials in bluegill (*n* = 4 individuals, five trials each for evasive and non-evasive prey; Kane and Higham 2020). Fish were similar in size across studies, and homologous kinematic traits included peak gape, time to opening, the velocity at peak gape, maximum velocity, time of maximum velocity (although previous work examined a greater portion of the strike event), acceleration at peak gape, and all three accuracy variables (these were not included in our prior PCA, Fig. 3). We found that both current treatments (healthy vs. damaged) produced an indistinguishable performance from prior non-evasive prey trials and that evasive prey trials were consistently different (Fig. 4, fish in panels A–C overlap in performance space). Since the published trials were collected in different years, filmed in different labs, and utilized fish from natural habitats (instead of a hatchery), the similarity suggests that our samples are indeed representative more broadly. Additionally, performance differences incurred by prior fin damage are less substantial than those incurred by evasive prey, and the minor damage and recovery experienced by our fish may be an ecologically minor challenge. However, this may not be the case if the damage is more extensive.

### Reduction of individual specialization

Examination of the PCA results (Fig. 3) indicates our most striking finding—fish may lose individualized patterns of prey capture behavior when exposed to fin damage, and individual capture success may not be the relevant metric for understanding the consequences of fin damage for bluegill. One explanation for this loss could be that constraints imposed by compensatory locomotor mechanisms (as in Higham et al. 2005a) could require individuals to perform more similarly when they have damaged fins. To explore the representation of this pattern, we again compared our data to previously published results in bluegill (Kane and Higham 2020).

Prominent individual effects were also discovered in prior work (Kane and Higham 2020), and this was further reflected in the qualitative increase in distance between individuals when capturing non-evasive prey (Fig. 4C and D), mimicking the current study. This comparison suggests that fin damage and capture of evasive prey likely represent parallel challenges to individualized strategies during prey capture, but that the pattern observed in damaged fish was less extreme than that observed for evasive prey, where differences in kinematics, accuracy, and success have been observed (Kane and Higham 2014). If the extent of damage impacts success in a continuous way (compared to presence vs. absence), then we expect that more severe damage may be required before differences in accuracy and success can be quantified. Future work should explore this relationship experimentally.

Individuals in our current study with previously damaged fins appear to lack the individual specialization apparent in healthy fish, and we suggest that further examination of fin damage during prey capture can add new insights into the higher-order emergent consequences for locomotor systems on dependent, integrated, ecologically relevant tasks. Bolnick et al. (2003) define an individual specialist as one whose trait variation encompasses a subset of the variation observed across the population. Under normal conditions, healthy fish may be individually specialized to a subset of the multivariate kinematic variation observed in the population (Fig. 3A). In stickleback (*Gasterosteus aculeatus*), this mechanism mediates the detrimental effects of increased intraspecific competition on individual fitness (Svanbäck and Bolnick 2005, 2007). Therefore, if bluegill or other fishes lose the ability to specialize in populations where it is favored, then a cost may be imposed on their ability to compete for prey in imperiled populations or resource-limited habitats. Additionally, fitness costs could compound in limited circumstances, such as during periods of low prey availability, cold temperatures, or hypoxia. We suggest that further exploration of the presence and degree of fin damage and its consequences for capture success, especially in competitive environments, is a fruitful area for future work.

## Conclusion

Our exploratory study suggests that fin damage as a result of capture using a cast net can impact variation in prey capture performance in hatchery-sourced bluegill, with potential consequences for success in extreme or competitive environments. In this way, the morphology and function of locomotor structures can have important, previously unidentified, emergent consequences on prey capture outcomes. However, the effects of the type, severity, and duration of damage may be relevant and remain to be examined. Future analyses are needed to further tease apart the effects of locomotor damage on integrated behaviors such as prey capture and should emphasize individual-level variation and its relationship to success. Ultimately, this line of questioning can be useful for considering the non-lethal effects of fin damage on other species, such as those in imperiled populations (Jelks et al. 2008), or for further understanding the indirect effects of catch-and-release fishing (Thompson et al. 2018; LaRochelle et al. 2022).

## Supplementary Material

obac050_Supplemental_File

## Data Availability

All videos and data representing analyzed points have been deposited in ZMAportal.org in the study “Bluegill fin damage prey capture” with permanent ID ZMA31.
