# Supplementary material for: Potential for Anthropogenic Fin Damage to Affect Individual Responses to Prey in Bluegill Sunfish (Lepomis macrochirus): A New Hypothesis for Kinematic Studies
Source: Integr Org Biol. 2022 Dec 16;4(1):obac050. doi: 10.1093/iob/obac050 (PMC9762888; doi:10.1093/iob/obac050)
Supplement: obac050_Supplemental_File [file obac050_supplemental_file.docx]

**SUPPLEMENTARY MATERIALS**

Effects of anthropogenic fin damage on prey capture kinematics and capture success in bluegill sunfish (*Lepomis macrochirus*)

Hannah E. Cohen^1^, William Ray^1^, Olivia H. Hawkins^2^, Emily A. Kane^2^

^1^: Department of Biology, Georgia Southern University, Statesboro, GA

^2^: Department of Biology, University of Louisiana at Lafayette, Lafayette, LA

**Table S1. Model selection of General Linear Mixed Models (GLMM) with and without standard length (SL) as a fixed effect for each kinematic variable.** Reported parameters for each model include AIC, BIC, log likelihood, deviance, Chi square (X^2^), degrees of freedom (df), and the p-value. The reduced model (Model 1) uses the following formula: *kinematic trait~Population + (1|Individual).* The full model (Model 2) uses the following formula: *kinematic trait~Population + SL + (1|Individual).* Population is the primary fixed effect that accounts for the observations belonging to healthy or damaged fish. Individual is included as a random effect to control for any influence of individual variation. A third model including the interaction of standard length and population as a fixed effect (SL:Population) was compared to Model 2 and found to not significantly differ therefore we proceeded with Model 2 as the full model. When an Analysis of Variance (ANOVA) returned a p-value below the cutoff (0.05), the full model was used for univariate analysis. Significance is denoted by an asterisk (*) and selected models are bolded.

**​​**

| **Kinematic variable** | **Model** | **AIC** | **BIC** | **Log likelihood** | **Deviance** | **X^2^** | **df** | **p-value** |
| --- | --- | --- | --- | --- | --- | --- | --- | --- |
| *Peak gape (cm)* | 1  **2** | -81.89  -90.72 | -73.64  -80.42 | 44.94  50.36 | -89.89  -100.72 | 10.84 | 1 | p < 0.0001* |
| *Peak protrusion (cm)* | 1  **2** | -158.49  -161.57 | -150.25  -151.27 | 83.244  85.788 | -166.49  -171.57 | 5.09 | 1 | 0.02* |
| *Protrusion velocity (cm/s)* | **1**  2 | 420.55  422.36 | 428.79  432.66 | -206.28  -206.18 | 412.55  412.36 | 0.19 | 1 | 0.66 |
| *Time of peak protrusion (ms)* | **1**  2 | 419.91  420.69 | 428.15  430.99 | -205.96  -205.34 | 411.91  410.69 | 1.22 | 1 | 0.27 |
| *Duration of mouth opening (ms)* | **1**  2 | 467.65  469.49 | 475.89  479.79 | 475.89  479.79 | -229.82  -229.75 | 0.16 | 1 | 0.69 |
| *Duration of mouth closing (ms)* | **1**  2 | 536.24  537.86 | 544.48  548.16 | -264.12  -263.93 | 528.24  527.86 | 0.38 | 1 | 0.54 |
| *Maximum body velocity (cm/s)* | **1**  2 | 433.39  435.39 | 441.63  445.69 | -212.69  -212.69 | 425.39  425.39 | 0.0001 | 1 | 0.99 |
| *Time of maximum velocity (cm/s)* | **1**  2 | 508.90  510.76 | 517.14  521.06 | -250.45  -250.38 | 500.90  500.76 | 0.14 | 1 | 0.70 |
| *Velocity at peak gape (cm/s)* | **1**  2 | 413.61  415.28 | 421.86  425.59 | -202.81  -202.64 | 405.61  405.28 | 0.33 | 1 | 0.57 |
| *Acceleration at peak gape (cm/s^2^)* | **1**  2 | 955.97  956.97 | 964.21  967.27 | -473.99  -473.48 | 947.97  946.97 | 1.00 | 1 | 0.32 |
| *Accuracy Index (AI)* | **1**  2 | 19.444  21.154 | 27.686  31.457 | -5.7222  -5.5772 | 11.444  11.154 | 0.29 | 1 | 0.59 |
| *Height to length ratio of ingested volume* | **1**  2 | -140.87  -139.44 | -132.63  -129.14 | 74.436  74.719 | -148.87  -149.44 | 0.57 | 1 | 0.45 |
| *Ingested volume (cm^3^)* | **1**  2 | 258.21  257.53 | 266.46  267.83 | -125.11  -123.77 | 250.22  247.53 | 2.68 | 1 | 0.10 |
| *Predator-prey distance at mouth opening (cm)* | **1**  2 | 77.44  78.70 | 85.68  89.00 | -34.72  -34.35 | 69.44  68.70 | 0.74 | 1 | 0.39 |

**Table S2. Comparison of General Linear Mixed Models (GLMM) with and without standard length (SL) as a fixed effect for each kinematic variable.** Reported parameters include the predictors and intercept, coefficient estimates, error, degrees of freedom (df), t values, p-values for the contribution of the predictor to the model. The reduced model (Model 1) uses the following formula: *kinematic trait~Population + (1|Individual).* The full model (Model 2) uses the following formula: *kinematic trait~Population + SL + (1|Individual).* Population is the primary fixed effect that accounts for the observations belonging to healthy or damaged fish. Individual is included as a random effect to control for any influence of individual variation. Significance is denoted by 2 asterisks (**) using a traditional cutoff of 0.05 or 1 asterisk (*) using a modified cutoff of 0.1.

| **Kinematic variable** | **Model** | **Predictors** | **Estimates** | **Error** | **df** | **t value** | **p-value by estimate** |
| --- | --- | --- | --- | --- | --- | --- | --- |
| *Peak gape (cm)* | 1  **2** | Intercept  Population  Intercept  Population  SL | 0.89  -0.0021  0.14  0.10  0.08 | 0.04  0.58  0.23  0.06  0.02 | 10.29  0.58  52.36  14.26  0.23 | 21.784  -0.036  0.63  1.58  3.38 | p < 0.0001**  0.97  0.53  0.14  p < 0.01** |
| *Peak protrusion (cm)* | 1  **2** | Intercept  Population  Intercept  Population  SL | 0.38  -0.08  0.12  -0.04  0.03 | 0.02  0.05  0.12  0.03  0.01 | 9.85  9.61  42.01  15.36  42.65 | 21.51  -3.08  1.04  -1.53  2.20 | p < 0.0001**  0.01**  0.30  0.15  0.03** |
| *Peak protrusion velocity (cm/s)* | **1**  2 | Intercept  Population  Intercept  Population  SL | 18.54  -1.37  11.60  -0.44  0.72 | 2.36  3.31  17.93  15.85  1.85 | 10.53  10.25  40.30  15.85  40.79 | 7.85  -0.42  0.65  -0.11  0.39 | p < 0.0001**  0.69  0.52  0.92  0.70 |
| *Time of peak protrusion (ms)* | **1**  2 | Intercept  Population  Intercept  Population  SL | -0.51  -2.16  19.40  -4.80  -2.10 | 3.43  4.84  18.62  5.43  1.90 | 9.75  0.62  53.81  13.40  54.63 | -0.15  -0.45  1.04  -0.88  -1.09 | 0.89  0.67  0.30  0.39  0.28 |
| *Duration of mouth opening (ms)* | **1**  2 | Intercept  Population  Intercept  Population  SL | 43.68  -4.68  29.57  -2.81  1.47 | 3.48  4.87  27.04  6.25  2.78 | 10.01  9.72  39.48  14.15  40.07 | 12.56  -0.96  1.09  -0.45  0.53 | p < 0.0001**  0.36  0.28  0.66  0.60 |
| *Duration of mouth closing (ms)* | **1**  2 | Intercept  Population  Intercept  Population  SL | 78.87  -17.00  109.72  -21.14  -3.21 | 7.19  10.10  49.87  12.24  5.13 | 10.34  10.11  46.41  15.42  47.30 | 10.97  -1.68  2.20  -1.73  -0.63 | p < 0.0001**  0.12  0.03**  0.10  0.53 |
| *Maximum velocity (cm/s)* | **1**  2 | Intercept  Population  Intercept  Population  SL | 26.73  1.39  28.26  1.18  -0.16 | 2.84  3.99  20.44  4.89  2.10 | 10.46  10.21  44.21  15.44  44.97 | 9.41  0.35  1.38  0.24  -0.08 | p < 0.0001**  0.74  0.17  0.81  0.95 |
| *Time of maximum velocity (cm/s)* | **1**  2 | Intercept  Population  Intercept  Population  SL | -22.95  -7.26  -7.14  -9.34  -1.65 | 3.66  5.09  34.65  7.13  3.57 | 9.33  8.95  23.16  12.78  23.00 | -6.28  -1.43  -0.21  -1.31  -0.46 | p < 0.0001**  0.19  0.84  0.21  0.65 |
| *Velocity at peak gape (cm/s)* | **1**  2 | Intercept  Population  Intercept  Population  SL | 19.66  2.28  11.94  3.31  0.80 | 2.45  3.44  17.17  4.09  1.77 | 10.43  10.18  43.47  14.91  44.24 | 8.04  0.66  0.70  0.81  0.45 | p < 0.0001**  0.52  0.49  0.43  0.65 |
| *Acceleration at peak gape (cm/s^2^)* | **1**  2 | Intercept  Population  Intercept  Population  SL | 62.52  -415.89  -1445.90  -209.10  156.2 | 164.79  229.13  1548.50  311.60  159.40 | 56.00  56.00  55.00  55.00  55.00 | 0.38  -1.82  -0.93  -0.67  0.98 | 0.71  0.07*  0.36  0.51  0.33 |
| *Accuracy Index (AI)* | **1**  2 | Intercept  Population  Intercept  Population  SL | 0.71  -0.03  0.466  0.01  0.03 | 0.05  0.07  0.49  0.98  0.05 | 56.00  56.00  55.00  55.00  55.00 | 13.91  -0.39  0.95  0.07  0.53 | p < 0.0001**  0.70  0.35  0.94  0.60 |
| *Height to length ratio of ingested volume* | **1**  2 | Intercept  Population  Intercept  Population  SL | 1.00  -0.01  0.88  0.003  0.01 | 0.02  0.02  0.14  0.03  0.01 | 10.41  10.06  33.60  14.06  33.84 | 62.28  -0.61  6.36  0.10  0.91 | p < 0.0001**  0.56  p < 0.0001**  0.92  0.37 |
| *Ingested volume (cm^3^)* | **1**  2 | Intercept  Population  Intercept  Population  SL | 5.86  0.32  -1.11  1.25  0.72 | 0.75  1.05  4.50  1.16  0.46 | 10.34  10.16  49.59  15.07  50.62 | 7.85  0.30  49.59  15.07  50.62 | p < 0.0001**  0.77  0.81  0.30  0.12 |
| *Predator-prey distance at mouth opening (cm)* | **1**  2 | Intercept  Population  Intercept  Population  SL | 1.68  -0.26  0.94  -0.16  0.08 | 0.12  0.17  0.93  0.21  0.10 | 10.34  10.06  39.72  15.48  40.21 | 13.72  -1.51  1.01  -0.75  0.81 | p < 0.0001**  0.16  0.32  0.46  0.42 |

**Table S3. Summary statistics for all kinematic variables (feeding, swimming, accuracy) by population.** Data are reported as mean ± SD (standard deviation) of all observations from healthy (n=28), and damaged (n=30) trials. Percent difference represents the change observed in the damaged fish compared to healthy fish.

| **Variable** | **Healthy** | **Damaged** | **% Difference** |
| --- | --- | --- | --- |
| **Kinematics** |  |  |  |
| *Peak gape (cm)* | 0.89 ± 0.15 | 0.89 ± 0.10 | 0 |
| *Peak protrusion (cm)* | 0.38 ± 0.08 | 0.30 ± 0.04 | -21.05 |
| *Peak protrusion velocity (cm/s)* | 18.58 ± 7.16 | 17.17 ± 10.23 | -7.59 |
| *Time of peak protrusion (ms)* | 0.36 ± 11.98 | -2.67 ± 7.34 | -841.67 |
| *Duration of mouth opening (ms)* | 43.29 ± 12.13 | 39.0 ± 14.21 | -9.91 |
| *Duration of mouth closing (ms)* | 78.36 ± 27.29 | 61.87 ± 22.17 | -21.04 |
| *Maximum body velocity (cm/s)* | 26.65 ± 7.58 | 28.11 ± 11.98 | 5.48 |
| *Time of maximum velocity (cm/s)* | -22.86 ± 19.33 | -30.20 ± 17.85 | 32.11 |
| *Velocity at peak gape (cm/s)* | 19.76 ± 7.92 | 21.94 ± 9.79 | 11.03 |
| *Acceleration at peak gape (cm/s^2^)* | 65.52 ± 806.27 | -353.38 ± 929.0 | -639.35 |
| **Accuracy** |  |  |  |
| *Accuracy Index (AI)* | 0.71 ± 0.34 | 0.69 ± 0.18 | -2.82 |
| *Height to length ratio of ingested volume* | 1. ± 0.6 | 0.99 ± 0.08 | -1.00 |
| *Ingested volume (cm^3^)* | 5.88 ± 2.63 | 6.18 ± 2.09 | 5.10 |
| *Predator-prey distance at mouth opening (cm)* | 1.67 ± 0.49 | 1.42 ± 0.43 | -14.97 |

**Table S4. Quadratic Discriminant Analysis coefficients for swimming and feeding kinematic variables.** Data used in the analysis include observations from 6 healthy (n= 28) and 6 damaged (n= 30) individuals regressed against standard length (SL). Coefficients are bolded and considered significant contributors to a PC when their magnitude is greater than | 1 |.

| **Kinematic variables** | **Discriminant Axis 1** |
| --- | --- |
| *Peak gape* | **1.338** |
| *Duration of mouth opening* | 0.035 |
| *Duration of mouth closing* | 0.021 |
| *Peak protrusion* | **3.627** |
| *Peak protrusion velocity* | 0.055 |
| *Time of peak protrusion* | 0.072 |
| *Velocity at peak gape* | -0.120 |
| *Maximum velocity* | 0.050 |
| *Time of maximum velocity* | 0.048 |
| *Acceleration at peak gape* | <0.0001 |

**Table S5. Loadings of principal components for feeding and swimming variables.** Data used in the analysis include observations from 6 healthy (n= 28 trials) and 6 damaged (n= 30 trials) individuals regressed against standard length (SL). Principal component (PC) loadings indicate the contribution of each variable to each PC. Loadings are bolded and considered significant contributors to a PC when their magnitude is greater than | 0.3 |.

| **Kinematic variable** | **PC 1** | **PC 2** |
| --- | --- | --- |
| *Peak gape* | 0.20 | **0.59** |
| *Duration of mouth opening* | **-0.42** | 0.21 |
| *Duration of mouth closing* | -0.28 | 0.07 |
| *Peak protrusion* | 0.03 | **-0.50** |
| *Peak protrusion velocity* | **0.42** | -0.15 |
| *Time of peak protrusion* | -0.21 | **-0.53** |
| *Velocity at peak gape* | **0.46** | 0.05 |
| *Maximum velocity* | **0.42** | -0.02 |
| *Time of maximum velocity* | 0.30 | -0.19 |
| *Acceleration at peak gape* | -0.07 | -0.03 |
| **Percentage of variance by component** | 38.9% | 15.8% |
| **Total percentage of variation** | 38.9% | 54.7% |
